# Value of Multiomics Over Clinical Risk Factors in Hypertension Prediction

**DOI:** 10.1161/HYPERTENSIONAHA.125.25358

**Published:** 2025-12-11

**Authors:** Matti Vuori, Matti O. Ruuskanen, Pekka Jousilahti, Veikko Salomaa, Li-Fang Yeo, Anni Kauko, Felix Vaura, Aki Havulinna, Yang Liu, Guillaume Méric, Michael Inouye, Rob Knight, Leo Lahti, Teemu Niiranen

**Affiliations:** Department of Internal Medicine, University of Turku and Turku University Hospital, Finland (M.V., L.-F.Y., A.K., T.N.).; Department of Public Health, The Finnish Institute for Health and Welfare (THL), Helsinki (M.O.R., P.J., V.S., A.H., T.N.).; Institute for Molecular Medicine Finland (FIMM), Helsinki University Institute for Life Sciences (HiLIFE), University of Helsinki (F.V., A.H.).; Department of Computing (M.O.R., A.H., L.L.), University of Turku, Finland.; InFLAMES Flagship (A.K.), University of Turku, Finland.; Cambridge Baker Systems Genomics Initiative, Department of Public Health and Primary Care (Y.L., M.I.), University of Cambridge, United Kingdom.; British Heart Foundation Cardiovascular Epidemiology Unit, Department of Public Health and Primary Care (Y.L., M.I.), University of Cambridge, United Kingdom.; Victor Phillip Dahdaleh Heart and Lung Research Institute (Y.L., M.I.), University of Cambridge, United Kingdom.; British Heart Foundation Centre of Research Excellence (Y.L., M.I.), University of Cambridge, United Kingdom.; Cambridge Baker Systems Genomics Initiative, Baker Heart and Diabetes Institute, Melbourne, Victoria, Australia (Y.L., G.M., M.I.).; Health Data Research UK Cambridge, Wellcome Genome Campus, Hinxton (Y.L., M.I.).; Department of Pediatrics (R.K.), University of California San Diego, La Jolla.; Department of Bioengineering (R.K.), University of California San Diego, La Jolla.; Department of Computer Science and Engineering (R.K.), University of California San Diego, La Jolla.; Center for Microbiome Innovation (R.K.), University of California San Diego, La Jolla.; Baker-Department of Cardiometabolic Health, University of Melbourne, Parkville, Victoria, Australia (G.M.).; Department of Infectious Diseases, School of Translational Medicine, Monash University, Melbourne, Victoria, Australia (G.M.).; Department of Cardiovascular Research, Translation and Implementation, La Trobe University, Melbourne, Victoria, Australia (G.M.).

**Keywords:** genome, hypertension, metabolome, microbiota, risk factors

## Abstract

**BACKGROUND:**

Several omics methods have been successfully used in hypertension prediction. However, the predictive ability of various multiomics data has not been compared in the same study sample, and it is unknown whether they provide additional predictive value over a good clinical risk factor score.

**METHODS:**

Clinical data augmented with modern multiomics methods (systolic blood pressure polygenic risk score, nuclear magnetic resonance metabolite profiling, and gut microbiota) were assessed in 2573 nonhypertensive participants of the FINRISK 2002 cohort. All combinations of these different methods were incorporated into cross-validated machine learning models to predict incident hypertension. Model performance of all combinations of these was assessed using the area under the curve (AUC). Information on incident hypertension was collected using nationwide healthcare register data.

**RESULTS:**

Over a mean follow-up of 18.0 years, 393 participants developed hypertension. Models that included the clinical and genetic data resulted in the highest mean AUC (0.735) compared with clinical risk factors alone (AUC=0.725). In the whole study sample, an SD increase in the polygenic risk score was associated with 29% (95% CI, 14%–46%) greater odds of incident hypertension after adjusting for clinical risk factors. Combining metabolome (AUC=0.709) or microbiota (AUC=0.720) data with clinical risk factors did not result in improved risk prediction.

**CONCLUSIONS:**

The best prediction combination model for incident hypertension was the clinical model augmented with a polygenic risk score. These data suggest that polygenic risk scores provide limited incremental value over clinical risk factors when assessing risk of incident hypertension.

**NOVELTY AND RELEVANCE**

**What Is New?**

We analyzed host genome, gut microbiota, and host metabolome data from 2573 nonhypertensive participants to elucidate the individual and joint predictive value of these omics methods for incident hypertension over a follow-up of 18 years.

**What Is Relevant?**

Using a multiomics approach, the best predictive value for hypertension was achieved with a combination of a polygenic risk score and clinical risk factors.

The study of multiomics adds to mechanistic understanding of hypertension even if it does not result in improved prediction.

**Clinical/Pathophysiological Implications?**

Although several omics markers have been associated with hypertension, their incremental predictive value over clinical risk factors may be limited.

The use of systolic blood pressure polygenic risk scores for improving hypertension risk assessment in clinical practice might be advocated in certain situations.

Recent technological progress has enabled the large-scale measurement of novel omics-based biomarkers through genotyping, gut microbiota sequencing, and mapping of the circulating metabolome. Large-scale genome-wide association analyses allow tracking disease-associated molecular changes down to the gene coding level, whereas metabolomics studies the effects of the downstream products in the circulation. The gut metagenome, or microbiome, is situated in-between these and is subject to numerous effects by the host and environmental factors. All these omics data have been separately linked with prevalent or incident hypertension.^1–3^ However, statistical associations with future hypertension risk do not necessarily imply improved risk prediction. In fact, these methods may provide only minor incremental improvements in predictive value over conventional clinical risk factors.^4^ In addition, the predictive values of various omics for incident hypertension have not been compared, and their joint predictive value is unknown. This information could be used to develop an optimal multiomics-based approach for hypertension risk assessment.

To estimate the individual and joint predictive value of the genome, gut microbiota, and circulating metabolome for hypertension, we analyzed data from 2573 nonhypertensive participants of a well-phenotyped and representative Finnish population cohort examined in 2002. In addition to assessment of clinical hypertension risk factors, the participants underwent genotyping, nuclear magnetic resonance (NMR) profiling of the circulating metabolome, and fecal microbiota sequencing. The participants were followed up for a mean period of 18 years after baseline, during which 393 participants developed incident hypertension.

## Methods

### Data Availability

The source code used to analyze the data is available at https://github.com/finrisk2002/article-2025-multiomics-and-hypertension. The code for calculating the systolic blood pressure (BP) polygenic risk scores (PRSs) is available at https://github.com/akauko/multi_ht. The metagenomic data are available from the European Genome-Phenome Archive (accession number EGAD00001007035). Because of the sensitive health information of included individuals, the other data sets analyzed during the current study are not public but are available through The Finnish Institute for Health and Welfare (THL) Biobank on submission of a research plan and signing a data transfer agreement (https://thl.fi/en/research-and-development/thl-biobank/for-researchers/application-process).

**Figure 1.**
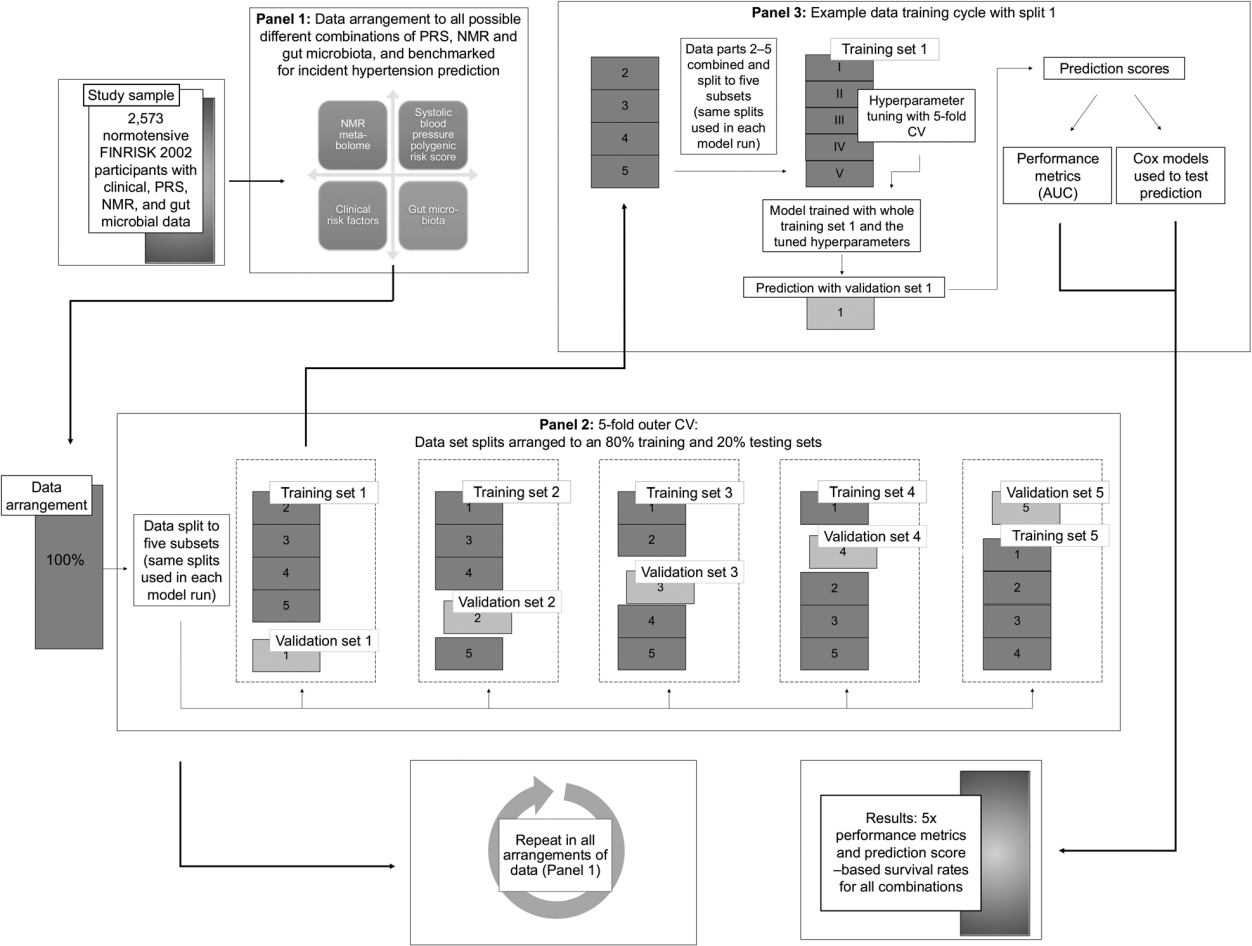
**Framework of the statistical and machine learning analyses.** AUC indicates area under the curve; CV, cross-validation; NMR, nuclear magnetic resonance spectroscopy; and PRS, systolic blood pressure polygenic risk score.

We assessed the extent to which hypertension risk prediction can be improved over conventional clinical risk factors by incorporating available multiomics data in the models using a combination of statistical and machine learning approaches (Figure 1). We combined baseline clinical, genotype, metabolome, and microbiota data to create a risk prediction model in its various combinations and assessed each combination model fit and risk discrimination performance.

### Study Sample

The FINRISK study is a series of population health surveys with age- and sex-stratified random sampling that have been organized at 5-year intervals since 1972 by THL, the Finnish Institute for Health and Welfare.^5^ The sample of this study was drawn from the FINRISK 2002 survey participants. In 2002, a total of N=13 500 individuals aged 25 to 74 years were invited to participate, and 8726 (64.6%) took part in the health examination.^6^ Altogether, 7231 of these participants gave fecal samples that were successfully sequenced. Out of these 7231 participants, we excluded (in this exact order) individuals with missing register linkage information or follow-up data (n=134), low microbiota sequencing quality (n=71), pregnant women (n=40), users of antibiotics in the 6 months preceding baseline investigation (n=894), participants with prevalent hypertension (n=2891), and individuals with missing genotype or metabolome data (n=628). After exclusions, 2573 nonhypertensive participants (at baseline) were included in the study sample and analyses.

### Baseline Examination

Medical history, including lifestyle information, was collected with a questionnaire before the baseline examination. Trained nurses measured BP 3× at 1-minute intervals after a 5-minute rest. Measurements were performed using a mercury sphygmomanometer and an appropriately sized cuff.^7^ Systolic and diastolic BP were defined as the first and the fifth Korotkoff sounds, respectively. BP was defined as the mean of the first 2 measurements. Height and weight were measured with standard methods. Venous blood sampling for the DNA, metabolome, lipids, and hemoglobin A1c was performed at the baseline examination, after a minimum of 4-hour fast.

### Clinical Data

The following clinical predictors were included in the models: age, sex, body mass index (BMI), mean arterial pressure, smoking status, leisure-time exercise, semifasting glucose, serum total cholesterol, serum triglycerides, hemoglobin A1c, and a Healthy Food Choices score. BMI was calculated as weight (kg) divided by height (m) squared. Mean arterial pressure was defined as diastolic BP+([systolic BP-diastolic BP]/3). Current smoking status was assessed with a 3-category variable based on self-report: (1) smokers: regular smoking for >1 year and smoking during the previous month; (2) ex-smokers: previous regular smoking, but quitting >1 month before the survey; and (3) never-smokers.^5^ Leisure-time exercise was defined using a self-reported 3-category variable: (1) no significant leisure-time exercise; (2) functional exercise (walking, cycling, or equivalent ) for at least 4 hours per week; (3) rigorous leisure-time exercise for at least 3 hours or more a week or regular training for competitive sports. The Healthy Food Choices score^8^ sums food propensity questionnaire responses to a score of 9 to 745, with a higher number indicating healthier food choices according to the 2014 Nordic Nutrition Recommendations dietary guidelines (ie, omnivorous Nordic diet rich in plants, fiber, and polyunsaturated fatty acids)^9^ based on self-report.

We defined prevalent hypertension at baseline using clinical and register data. Individuals with a systolic BP ≥140 mm Hg, diastolic BP ≥90 mm Hg, self-reported use of antihypertensive medication, or a register-based diagnosis of hypertension were considered to have prevalent hypertension (see Outcome Variable for the definition of register-based hypertension).

### Omics Data

We incorporated (1) clinical data with 3 different omics data sets in the predictive models: (2) the host genome; (3) the circulating metabolome; and (4) the fecal microbiota, each one separately or augmented with one to three combinations of (1) to (4).

For the host genetic data, genotyping and quality control have been previously described in detail.^10^ In short, FINRISK participants were genotyped using Illumina arrays. Sample-wise quality control excluded those with ambiguous sex, >5% missingness, ±4 SD heterozygosity, and non-European ancestry, while single-nucleotide polymorphism (SNP)–wise quality control excluded SNPs with >2% missingness, <1×10^−6^ Hardy-Weinberg equilibrium *P* value, and <3 (zCall) or <10 (GenCall) minor allele count. Genotype data were then prephased using Eagle2 (version 2.3) and imputed against the Finnish-specific SISu (Sequencing Initiative Suomi) v3 reference panel.^11^ PRS weights for a systolic BP PRS were downloaded from the PRS catalogue (ID: PGS000913). These weights are based on a genome-wide association study summary statistics for systolic BP from the UK Biobank (data field 4080, rank normalized) and computed with PRS-CS (polygenic risk score via continuous shrinkage)^12^ using default parameters and a European linkage disequilibrium reference panel from the 1000 Genomes Project.^13,14^ The PRS was based on 1098015 genetic variants common in the linkage disequilibrium reference panel and FINRISK. The PRS was computed in FINRISK with PLINK 1.9 and normalized across all FINRISK participants.

The circulating metabolome was assessed from peripheral blood using a 1H-NMR (hydrogen-1 nuclear magnetic resonance spectroscopy) Nightingale panel^15^ consisting of 226 circulating metabolites.^16^ The Nightingale method can be used to calculate absolute concentrations for chosen metabolites. A list of these metabolite measures is provided in Table S1. Each 96-well plate contained 2 quality control samples for monitoring the performance of the system. Metabolites were quantified by regression models from the spectra. Individual metabolites underwent statistical quality control and checks against an extensive reference database.

Gut microbiota profiling was performed using shallow metagenomic sequencing as previously described,^17^ with an average of 1 million reads per sample. We performed taxonomic mapping using Greengenes2.^18^ We excluded rare bacterial species that were present in <5% of the samples at the 0.1% relative abundance detection threshold, resulting in 258 species that were included in the analyses. We transformed the raw taxa counts with centered log-ratio, using a pseudocount of 1 before analysis.

### Outcome Variable

Incident hypertension was defined using data from nationwide Hospital Discharge, Causes of Death, and Drug Reimbursement registers.^19^ Having a hypertension-related *International Classification of Diseases*, *Eighth Revision* (401), *International Classification of Diseases*, *Ninth Revision* (401), or *International Classification of Diseases*, *Tenth Revision* (I10) code in the Hospital Discharge or Causes of Death registers was considered hypertension. In addition, a special reimbursement code 205 for hypertension or a thiazide diuretic or dihydropyridine calcium channel blocker purchase (ATC codes C03A, C03BA11, C03EA01, C07B, C09BA, C09DA, C07FB, C08CA, C08GA, C09BB, C09BX01, C09BX03, C09BX04, C09DB, C09DX, and C09XA) in the Drug Reimbursement register was considered hypertension. Follow-up data for hypertension were available until December 31, 2021.

### Statistical Analyses

Characteristics of individuals (1) with and without incident hypertension and (2) who took and did not take part in the fecal sampling were compared using the Wilcoxon rank-sum test for continuous variables and the Fisher test for categorical variables.

First, the clinical and omics data sets were benchmarked for hypertension prediction by using models that included all 15 different combinations of the clinical risk factors, gut metagenomics data, blood metabolomics data, and the PRS as predictors (see the framework in Figure 1). The outcome variable was incident hypertension. Modeling was performed using XGBoost (R package xgboost 1.7.3.1), a gradient boosting tree machine learning algorithm.^20^ We chose the XGBoost algorithm for the benchmarking, because it has performed well in previous studies in the same cohort.^17,21,22^ Each data set combination was tested in 5 cross-validated models with an 80% training and 20% testing set split. The same splits were consistently used across all models to maintain comparability across the benchmark. The splits do not overlap so that for each data combination, each study participant was included 4 times in the training data, and once in the testing data.

For each of the 5 cross-validation splits, the genetic principal components were calculated separately in the 80% training set, and the data in the 20% testing set were then fitted onto these components. This was performed to prevent data leakage between the training and testing splits, which would happen if the principal components based on genotyping were calculated only once for the whole cohort data. The first 20 genetic principal components were then used together with the PRS in all model runs that included genomic data, to control for population structure.

During model construction, the hyperparameters of each XGBoost model were first optimized with the R package mlrMBO 1.1.51 using an inner 5-fold cross-validation nested inside each of the 80% training sets (of the outer 5-fold cross-validations).^23^ Each model with the optimal hyperparameters was then trained with the full (80%) training data of the respective outer split. Finally, to be able to compare all models across the data set, we calculated the area under the curve (AUC) using predictions in each model’s respective test data, that is, the left-out 20% test set of the respective outer split.

To ease the interpretation of results with a more conventional statistical approach, the individual variables in the best-performing prediction model (clinical variables plus genome) were included in a Cox survival model in the whole study sample. We also assessed the increase in the Cox model goodness-of-fit when the PRS was added into a model with clinical variables using the likelihood ratio χ^2^ test. For these models, continuous variables were normalized to a mean of 0 and an SD of 1.

Several sensitivity analyses were performed using Cox modeling in the best-performing model. First, we assessed the predictive value of a model that included only age, mean arterial pressure, BMI, and exercise as the independent variables. Second, we included interaction terms between the PRS and all clinical variables in separate models for each clinical variable to assess whether the association between PRS and incident hypertension is dependent on the clinical characteristics of the participants. Third, we analyzed the data separately in men and women to assess if there are sex differences in the risk factors. Fourth, to enable comparison of risk discrimination between the machine learning model and the conventional Cox model, we produced calibration plots and performed a 5-fold cross-validation of the Cox model using the R package riskRegression.^24^ Fifth, we used a definition of incident hypertension that was solely based on register-based diagnoses and drug reimbursement rights while excluding the drug purchase-based cases. Sixth, to evaluate the incremental value of adding PRS to the clinical risk factors, changes in continuous integrated discrimination index, categorical integrated discrimination index (<10%, 10%–<20%, and ≥20% risk categories), and net reclassification improvement were calculated using the R package PredictABEL.^25^ For these models, we also assessed the agreement between predictions and observations in deciles of the predicted risk. All analyses were conducted using R version 4.2.2.^26^

## Results

The characteristics of the 2573 individuals in the study sample are presented in the Table. The mean age of the sample was 44.3±12.0 years, and 60.3% were women. The FINRISK 2002 participants who took part in the fecal sampling were older and, as a result, had higher BMI, higher mean arterial pressure, and higher cholesterol levels compared with individuals who did not participate (Table S2). In addition, participants were more likely to be women and nonsmokers compared with nonparticipants (Table S2). A total of 393 participants developed hypertension over a mean follow-up of 18.0 years. A total of 251 cases were identified by register-based diagnoses, and 275 cases were identified based on purchase of antihypertensive medications. Individuals with incident hypertension exercised less, were older, and had higher BMI, higher mean arterial pressure, higher plasma glucose, higher plasma total cholesterol, higher PRS, and lower plasma HDL (high-density lipoprotein) cholesterol compared with individuals who did not develop hypertension (Table).

**Table.**
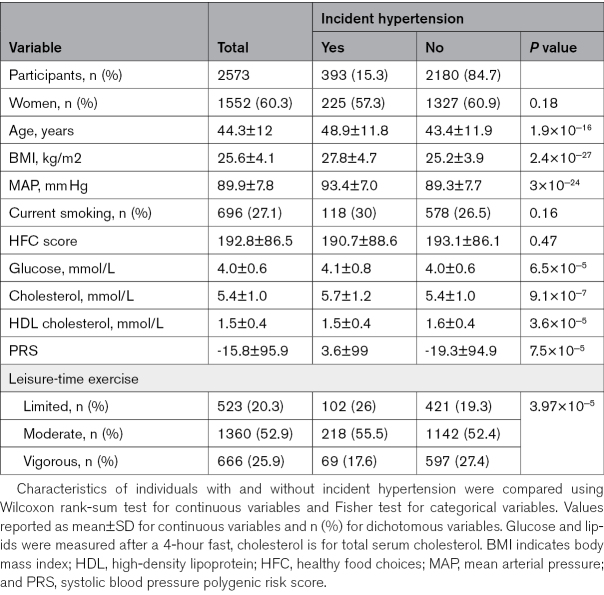
Study Sample Characteristics

**Figure 2.**
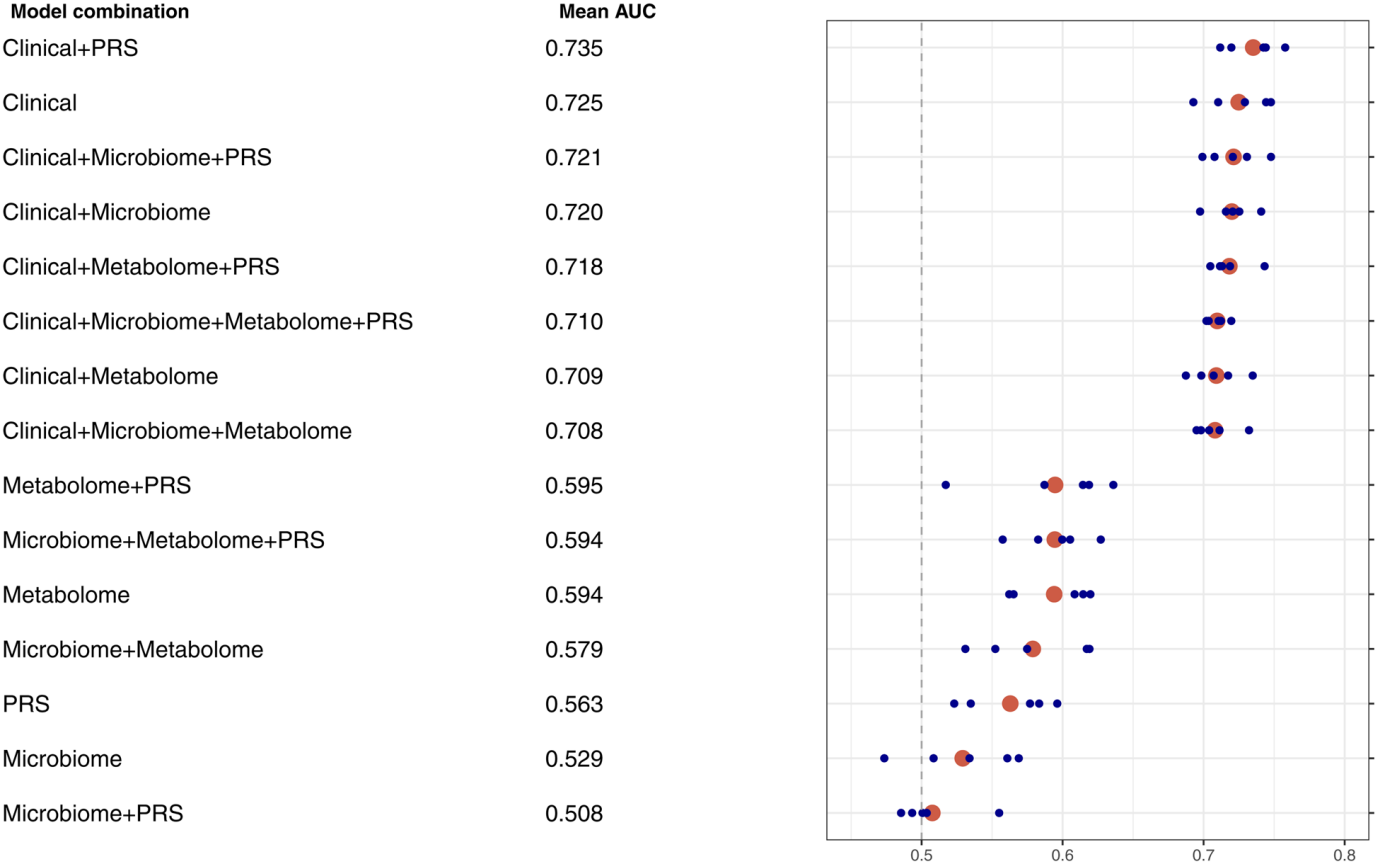
**Performance of clinical and omics data as predictors of incident hypertension.** Each of the 5 iterations is depicted as a small blue dot. A calculated mean of the 5 iterations is shown as an orange dot. The dashed line represents the reference level of 0.5 (equivalent to a coin toss). The clinical variables included age, sex, body mass index, mean arterial pressure, smoking status, leisure-time exercise, semifasting glucose, serum total cholesterol, serum triglycerides, hemoglobin A1c, and a Healthy Food Choices score. AUC indicates area under the curve; PRS, systolic blood pressure polygenic risk score.

**Figure 3.**
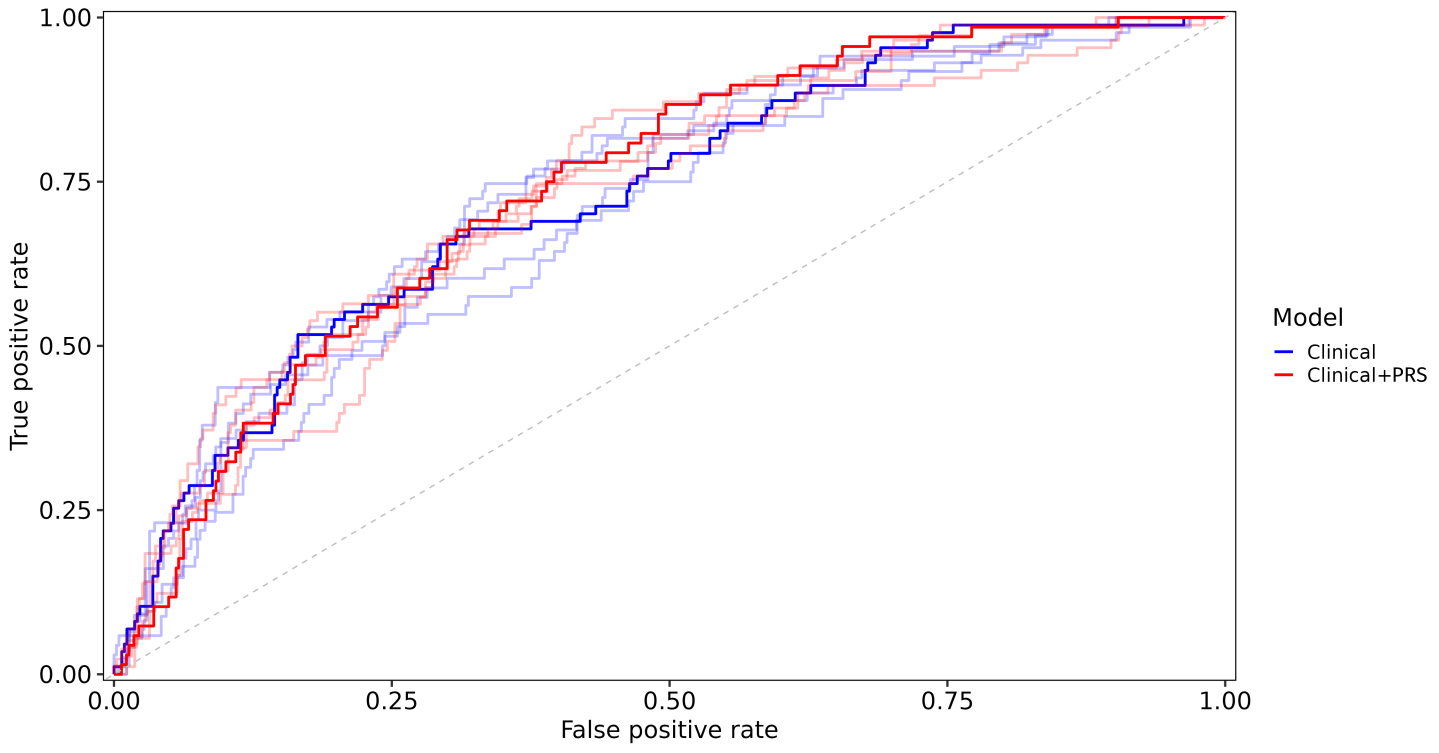
**The receiver operating characteristic curves for all 5 iterations in the 2 best-performing models: (1) clinical variables and (2) clinical variables and genomic data.** The curve with the area under the curve (AUC) closest to the model’s median AUC value from both models is bolded. PRS indicates systolic blood pressure polygenic risk score.

The performance of the various models for predicting incident hypertension is reported in Figure 2 and Table S3. The model, including the clinical and PRS together, resulted in the highest mean AUC value of 0.735, whereas clinical data alone provided the second-best value of 0.725. The receiver operator curves for all 5 iterations of the clinical and clinical plus PRS models are depicted in Figure 3. The predictive value of single-omics models ranged from stool metagenomics (AUC=0.529) to PRS (AUC=0.563) and metabolome (AUC=0.594). Models without clinical data consistently performed worse than models with clinical data (Figure 2). The microbiota and metabolome data did not improve risk prediction over clinical variables.

**Figure 4.**
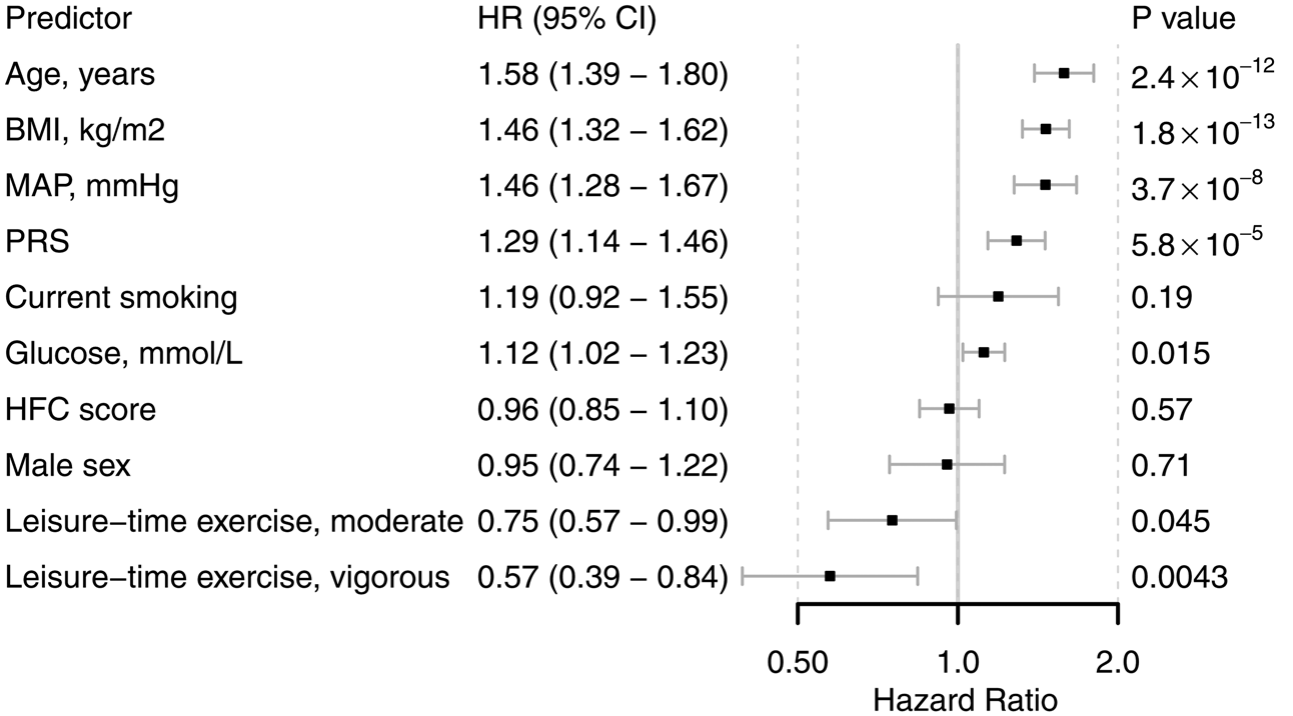
**Association between individual predictors included in the best-performing model (the clinical data with polygenic risk scores) and incident hypertension.** All variables were measured at baseline and simultaneously included in the model. Hazard ratios (HRs) for continuous variables are reported per 1-SD increase and adjusted for principal components 1 to 20. Plasma glucose is a 4-hour semifasting value. Exercise variables are compared with the lowest class (limited). BMI indicates body mass index; HFC, healthy food choices; MAP, mean arterial pressure; and PRS, systolic blood pressure polygenic risk score.

For deeper understanding of the best-performing prediction model, we assessed the association between individual risk factors and incident hypertension from the model with the highest AUC (clinical variables plus PRS; Figure 2) in the whole study sample using a multivariable Cox model (Figure 4). Of the variables included in the model, age (*P*=2.38×10^−12^), BMI (*P*=1.77×10^−^^13^), mean arterial pressure (*P*=3.72×10^−^^8^), PRS (*P*=5.79×10^−^^5^), and semifasting plasma glucose (*P*=0.015) were related to an increased risk of incident hypertension. More strenuous leisure-time exercise was associated with a lower risk of incident hypertension (*P*=3.97×10^−^^5^). The C statistic of this model was 0.759, whereas it was 0.749 for the model without the PRS. Adding the PRS in the model with clinical factors significantly improved the model fit (χ^2^=16.4, *P*=5.08×10^−^^5^ for difference). Addition of the PRS in a model that included the clinical risk factors significantly improved continuous net reclassification improvement (0.14 [95% CI, 0.02–0.26]; *P*=0.02) and integrated discrimination index (0.006 [95% CI, 0.0005–0.01]; *P*=0.03), but not categorical net reclassification improvement (*P*=0.09). Model calibration for both models was satisfactory, and no major differences in model calibration were observed, as indicated by the identical Brier scores (Figure S1).

In sensitivity analyses, while applying Cox modeling in the best-performing model (Figure 4), we observed no significant interaction between the PRS and the clinical variables (*P* for interaction for all ≥0.12). After 5-fold cross-validation, we observed a mean C statistic of 0.749, which was somewhat higher than what was achieved with the machine learning approach. With only age, mean arterial pressure, BMI, and exercise included as predictors, the Cox model had a C statistic of 0.721. When the outcome variable was based solely on register-based diagnoses and drug reimbursement rights, the C statistic was 0.791. When the best-performing Cox model was analyzed separately in men and women, the differences in statistical significance were minor (Table S4). Smoking was significantly associated with incident hypertension in women (*P*=0.022, but not in men (*P*=0.62).

## Discussion

In this study comprising 2573 Finnish individuals, we examined the individual and combined predictive performance of clinical risk factors and omics data for incident hypertension. We observed that the best predictive performance was achieved by combining clinical variables with the host PRS, with the latter providing limited improvement in risk discrimination and continuous, but not categorical, net reclassification improvement over clinical risk factors. The gut microbiota and the circulating metabolome did not improve risk prediction.

BP has an estimated heritability of 30% to 60%.^27^ To date, >2100 SNPs have been associated with BP.^28^ In contrast to single SNPs with very small effect sizes, PRSs provide an effective way to combine information from several SNPs into a single variable that represents the genetic risk of disease. Modern PRSs with over 1 million SNPs have been shown to improve hypertension prediction over clinical risk factors.^14^ One of the main advantages of the genome over other omics data is that it remains relatively unchanged over the lifetime of an individual.^29^ The current study demonstrates that a BP PRS offers incremental predictive value for hypertension not only over clinical risk factors, but also over other omics data.

The gut microbiota has been previously cross-sectionally linked with several metabolic and gastrointestinal conditions.^17^ Smaller human studies have demonstrated that hypertensive individuals display reduced alpha diversity, increased *Firmicutes*/*Bacteroidetes* ratio, and enrichment of taxa such as *Prevotella* and *Klebsiella* in their gut microbiome.^2,30^ Metabolomic profiling has further demonstrated altered levels of short-chain fatty acids, bile acids, and trimethylamine-*N*-oxide in hypertensive cohorts.^31^ In two larger separate population studies from Finland and the Netherlands, several microbial taxa have been cross-sectionally linked with hypertension.^32,33^ In the Dutch cohort, participants with lower BP had higher abundances of several short chain fatty acid producing microbes, but surprisingly, they had lower fecal levels of these fatty acids.^33^ Despite these previously observed associations, the incremental predictive value of the gut microbiota for incident hypertension was limited in our study. Previous cross-sectional studies may suffer from reverse causality, as hypertension, hypertensive medications, and dietary changes may all affect the microbiota. Therefore, the gut microbiota composition may not be a good risk factor for hypertension, and additional functional data are needed.^34^ In addition, unlike the host genome, the gut microbiota composition, albeit relatively stable in adulthood, can change over time in response to lifestyle alterations and the onset of certain other diseases.^34,35^ Thus, a significant part of the variation in microbiota could already be captured by clinical hypertension risk factors, diminishing its additive value in hypertension risk prediction models. Still, untargeted metabolomics could be used to provide complementary and mechanistic insights into the relationships between gut dysbiosis and hypertension by measuring the products of gut microbial metabolism in the stool and in the circulation.^36^

Metabolic alterations in multiple biochemical pathways can be seen as the first signs of the *circulus vitiosus* leading to hypertension. NMR profiling of the circulating metabolome has been successfully used to discover metabolites associated with the future onset of hypertension.^3,37–39^ These studies have highlighted the complexity of hypertension pathophysiology, as the metabolites identified are involved in multiple metabolic pathways, namely amino acid metabolism, fatty acid metabolism, oxidative stress, and inflammation.^3,37–39^ However, as with the gut microbiota, circulating metabolites are at the same time connected with environmental and clinical factors (eg, diet and medication use) and genetics.^40^ Therefore, it may be difficult to separate these components and their predictive value for hypertension from each other. Additionally, although mass spectrometry data may be preferable over NMR-based metabolomics, some dietary correlates and byproducts of microbiota metabolism, such as acetate, cholesterol, phosphatidylcholines, and triglycerides,^29^ are also available in NMR metabolite data. In our study, the circulating metabolome was slightly more strongly associated with incident hypertension than the gut microbiota. Our findings, therefore, highlight that functional metabolic profiling of blood or fecal samples could potentially provide a more robust risk prediction for hypertension compared with microbial composition.

The previous research on the association between multiomics and hypertension has been limited. The only earlier evidence, by Drouard et al,^41^ comes from the Finnish Twin Cohort and Young Finns study, where the associations of clinical, genetic, epigenetic, metabolomic, and transcriptomic data with BP were assessed. In this study, cross-sectional analyses were performed in the Finnish Twin Cohort Study using machine learning methods, and prospective analyses in the Young Finns Study. The authors observed that a combination of clinical risk factors, metabolome, and transcriptome data provided the best predictive power for elevated systolic BP. As in our study, the combination of clinical and genetic data provided the best predictive value for hypertension. However, there are methodological differences between our study and the study by Drouard et al.^41^ In our study, semifasting glucose, serum total cholesterol, serum triglycerides, and hemoglobin A1c were included in the clinical risk factors, whereas Drouard et al^41^ included these variables among the metabolic variables. In addition, the genetic risk scores used in Drouard et al^41^ included only selected SNPs with *P* values under 1×10^−5^. To our knowledge, ours is the first comparative study on modern omics methods for predicting incident hypertension in a prospective setting. The current available evidence suggests that combining clinical risk factors with PRSs could be the optimal approach for hypertension prediction—a good clinical risk score being of most benefit, with PRS adding improved prediction.^14^

In addition to comparing various types of omics data, comparing the bioinformatic and other computational methods is essential to increase the accuracy of hypertension prediction in the long run. The optimization of incident predictions using machine learning is a rapidly developing area of research.^40^ Not only different omics methods, but also different feature selection strategies and choice of the machine learning method may make a difference. Recently, a DREAM (Dialogue for Reverse Engineering Assessment and Methods) challenge investigated the optimal out-of-the-box prediction methods for incident heart failure.^42^ Although the incorporation of microbiome data did not increase the prediction performance over conventional risk factors, it provided important biological insights. However, instead of biological insights, our study focused solely on comparing the predictive performance of multiple omics data using a simple but functional machine learning model that has been shown to have a good performance in such tasks.^43^ In the current study, evaluating the effect of model architecture was therefore beyond our scope. Furthermore, feature selection is similarly important to increase the performance of incident disease modeling.^42^ In our analyses, we reduce the dimensionality of the data by using a PRS as a surrogate for the genotype and by limiting the microbiome analyses to the 258 most prevalent and abundant species. In addition, XGBoost outperforms many other methods for handling high-dimensional data.^44^ Due to the high dimensionality of the data used in this study, we initially opted to use a machine learning approach for data analysis. However, no high-dimensional data, such as the metabolome or the microbiome, were included in the best-performing model in the end. Therefore, traditional Cox regression modeling, which also takes time-to-event into account, provided an arithmetically greater C statistic than a machine learning approach (0.749 versus 0.735). These results highlight that machine learning may not provide additional value over conventional statistics when the number of predictor variables is low.

In addition to strengths, such as a randomly sampled population cohort, the long-term follow-up, standardized measurement of predictor variables to reduce heterogeneity, and the availability of several high-quality omics data modalities, this study has some limitations. First, we used a study sample drawn from a relatively homogenous population of Finnish individuals. Our results may not, therefore, be generalizable to other ethnicities and populations. Second, the diagnosis of incident hypertension in this study is based on register data, which underestimates the true incidence of hypertension during follow-up, as up to 50% of individuals with hypertension are unaware of having high BP, even in high-income countries.^45^ As demonstrated by our sensitivity analysis, model discrimination can be slightly increased by using only *International Classification of Diseases*, *Tenth Revision* codes and drug reimbursement rights, instead of combining these data with antihypertensive drug purchases, when defining hypertension. However, this approach leads to detecting only cases of hypertension that are more severe, which is needed for special drug reimbursement in Finland. In addition, as we are mainly assessing the relative risks of hypertension, the definition of hypertension has little effect on the ranking of the predictors. Third, although NMR metabolomics can distinguish large high-density metabolites (mostly lipids), it is not as sensitive to smaller molecules as mass spectrometry-based techniques.^46^ Fourth, we were limited to single baseline measurements of the clinical and omics risk factors, which do not account for chronic exposures to risk factors. Although the genome remains nearly unchanged after conception, the metabolome and the microbiome can change over time. However, both are fairly stable over the long term, with intraclass correlations ranging between 0.6 and 0.9.^47–49^ Fifth, risk prediction always depends on the predictor variables included in the model, and some hypertension risk factors may remain unknown at this time. Finally, family history of hypertension was not available in our study.

## Perspectives

Using a multiomics machine learning approach, supported by conventional statistics, we demonstrate that a systolic BP PRS provides small incremental predictive value over clinical risk factors for predicting future hypertension. In contrast, NMR metabolome and gut microbiota provided no incremental predictive value. Although the optimal method for detecting hypertension would be frequent BP measurements in everyone over the life course, this may not always be the case in clinical practice. The combined use of clinical risk factors and PRSs for hypertension risk assessment could potentially be used in niche situations to motivate individuals at high risk and to optimally target prevention strategies. Our results could also be used to inform the use of whole genome sequencing and personalizing care at a younger age for a more holistic approach to health, as opposed to obtaining genetic information to solely predict future hypertension.

## ARTICLE INFORMATION

### Sources of Funding

T. Niiranen was supported by the Research Council of Finland (grants 321351 and 354447), the Sigrid Jusélius Foundation, and the Finnish Foundation for Cardiovascular Research. M.O. Ruuskanen was supported by the Research Council of Finland (338818) and the Finnish Cultural Foundation. L-F. Yeo was supported by the European Union´s Horizon Europe Framework program for research and innovation 2021 to 2027 under the Marie Skłodowska-Curie grant agreement No 101126611. L. Lahti and M.O. Ruuskanen were supported by the European Union’s Horizon 2020 research and innovation program (grant 952914). This work was supported by core funding from the British Heart Foundation (BHF) (RG/18/13/33946, RG/F/23/110103), The National Institute for Health and Care Research (NIHR) Cambridge Biomedical Research center (BRC-1215–20014, NIHR203312), Cambridge BHF center of Research Excellence (RE/18/1/34212, RE/18/1/34212), as well as by Health Data Research UK, which is funded by the UK Medical Research Council, Engineering and Physical Sciences Research Council, Economic and Social Research Council, Department of Health and Social Care (England), Chief Scientist Office of the Scottish Government Health and Social Care Directorates, Health and Social Care Research and Development Division (Welsh Government), Public Health Agency (Northern Ireland), British Heart Foundation and Wellcome. The views expressed are those of the authors and not necessarily those of the National Health Service, the NIHR, or the Department of Health and Social Care.

### Disclosures

T. Niiranen has received speaking honoraria from AstraZeneca and Orion Finland. R. Knight is a scientific advisory board member and consultant for BiomeSense Inc, has equity, and receives income. He is a scientific advisory board member and has equity in GenCirq; is a consultant for DayTwo and receives income; has equity in and acts as a consultant for Cybele; is a cofounder of Biota Inc and has equity; is a cofounder of Micronoma and has equity; and is a scientific advisory board member. The terms of these arrangements have been reviewed and approved by the University of California, San Diego, in accordance with its conflict-of-interest policies. The other authors report no conflicts.

### Supplemental Material

Tables S1–S4

Figure S1

## Supplementary Material


