# Supplementary material for: Value of Multiomics Over Clinical Risk Factors in Hypertension Prediction
Source: Hypertension. 2025 Dec 11;83(2):e25358. doi: 10.1161/HYPERTENSIONAHA.125.25358 (PMC12822772; doi:10.1161/HYPERTENSIONAHA.125.25358)

**The Additive Value of Multiomics over Clinical Risk Factors**
**in Predicting Incident Hypertension**

**SUPPLEMENTARY APPENDIX**

Matti Vuori^a^, M.D., Ph.D., Matti O. Ruuskanen^b,d^, Ph.D.,
Pekka Jousilahti^b^, M.D., Ph.D., Veikko Salomaa^b^, M.D., Ph.D., Li-Fang Yao^a^, Ph.D., Anni Kauko, Ph.D.^a,r^, Felix Vaura^c^, M.D., Ph.D., Aki Havulinna^b-d^, D.Sc. (Tech.), Yang Liu, Ph.D.^e-j^, Guillaume Méric, Ph.D.^f,o-q^, Michael Inouye^e-j^, Ph.D.,
Rob Knight ^k-n^, Ph.D, Leo Lahti^d^, D.Sc. (Tech.), Teemu Niiranen^a,b^, M.D., Ph.D.

^a^ Department of Internal Medicine, University of Turku and Turku University Hospital, Turku, Finland

^b^ Department of Public Health, The Finnish Institute for Health and Welfare (THL), Helsinki, Finland

^c^ Institute for Molecular Medicine Finland (FIMM), HiLIFE, University of Helsinki, Helsinki, Finland

^d^ Department of Computing, University of Turku, Turku, Finland

^e^ Cambridge Baker Systems Genomics Initiative, Department of Public Health and Primary Care, University of Cambridge, Cambridge, UK

^f^ Cambridge Baker Systems Genomics Initiative, Baker Heart and Diabetes Institute, Melbourne, Victoria, Australia

^g^ British Heart Foundation Cardiovascular Epidemiology Unit, Department of Public Health and Primary Care, University of Cambridge, Cambridge, UK

^h^ Victor Phillip Dahdaleh Heart and Lung Research Institute, University of Cambridge, Cambridge, UK

^I^ Health Data Research UK Cambridge, Wellcome Genome Campus, Hinxton, UK

^j^ British Heart Foundation Centre of Research Excellence, University of Cambridge, Cambridge, UK

^k^ Department of Pediatrics, University of California San Diego, La Jolla, CA, USA

^l^ Center for Microbiome Innovation, University of California San Diego, La Jolla, CA, USA

^m^ Department of Bioengineering, University of California San Diego, La Jolla, CA, USA

^n^ Department of Computer Science and Engineering, University of California San Diego, La Jolla, CA, USA

^o^ Baker-Department of Cardiometabolic Health, University of Melbourne, Parkville, VIC 3010, Australia;

^p^ Department of Infectious Diseases, School of Translational Medicine, Monash University, Melbourne, Victoria 3004, Australia;

^q^ Department of Cardiovascular Research, Translation and Implementation, La Trobe University, Melbourne, Victoria; Australia

^r^ InFLAMES Flagship, University of Turku, Finland.

**Short title:** Multiomics in hypertension

**Correspondence to:** 
Matti Vuori A, Department of Internal Medicine, Turku University Hospital, Kiinanmyllynkatu 4-8, 20520 Turku, Finland. Email: makvuo@utu.fi

**Supplementary Tables**

**Supplemental Table S1.** A list of the 226 circulating metabolites measured with the 1H-NMR spectroscopy.

| **14 Lipoprotein subclasses *** | 6 VLDL subclasses XXL-XS |
| --- | --- |
|  | IDL |
|  | 3 LDL subclasses L-S |
|  | 4 HDL subclasses XL-S |
| **Ketone bodies (mmol/i)** | ﻿﻿Acetate |
|  | ﻿﻿Acetoacetale |
|  | ﻿﻿3-hydroxybutyrate |
| **Glycolysis related metabolites (mmol/i)** | Lactate |
|  | ﻿﻿Pyruvate |
|  | Citrate |
|  | Glycerol |
| **Inflammation (mmol/i)** | Glycoprotein acetyls (mainly α1-acid glycoprotein) |
| **Fatty acids and saturation** | ﻿﻿Total fatty acids |
|  | ﻿﻿Estimated degree of unsaturation |
| **Fatty acids (mmol/l and % of total fatty acids)** | ﻿Omega-3 fatty acids |
|  | Omega-6 fatty acid |
|  | Polyunsaturated fatty acids |
|  | Monounsaturated fatty acids; 16:1, 18:1 |
|  | ﻿Saturated fatty acids |
|  | ﻿Docosahexaenoic acid; 22:6 |
|  | ﻿Linoleic acid; 18:2 |
| **Amino acids (mmol/l)** | Alanine |
|  | Glutamine |
|  | Glycine |
|  | Histidine |
| Branched-chain amino acids | Isoleucine |
|  | Leucine |
|  | Valine |
| Aromatic amino acids | Phenylalanine |
|  | Tyrosine |
| **Cholesterol (mmol/l)** | VLDL cholesterol |
|  | LDL cholesterol |
|  | HDL cholesterol |
|  | HDL_2_ cholesterol |
|  | HDL_3_ cholesterol |
|  | Cholesterol |
|  | Free cholesterol |
|  | Esterified cholesterol |
|  | Remnant cholesterol |
| **Apolipoproteins (g/l)** | ApoA-I |
|  | ApoB |
| Apolipoproteins ratios | Ratio of ApoB to ApoA-I |
| **Fluid balance** | Creatinine (mmol/l) |
|  | Albumin (signal area) |
| **Glycerides & phospholipids (mmol/I)** | ﻿﻿VLDL triglycerides |
|  | ﻿﻿LDL triglycerides |
|  | HDL triglycerides |
|  | ﻿﻿Phosphoglycerides |
|  | Phosphatidyicholine and other cholines |
|  | Sphingomyelins |
|  | Total cholines |
| Glycerides & phospholipids ratios | ﻿﻿Ratio of diglycerides to triglycerides |
|  | ﻿﻿Ratio of triglycerides to phosphoglycerides |

* 12 lipid measures for each subclass–esterified cholesterol, free cholesterol, triglycerides, phospholipids, total cholesterol (mmol/l and % of total lipids for all), total lipids (mmol/I) and particle concentration (μmol/I)–and the mean diameter (nm) of VLDL, LDL and HDL particles are also calculated.

Abbreviations: VLDL, very low-density lipoprotein cholesterol; LDL, low-density lipoprotein cholesterol; IDL, intermediate-density lipoprotein cholesterol, HDL: high-density lipoprotein cholesterol; ApoA-I, apolipoprotein A-I; ApoB, apolipoprotein B.

**Supplemental Table S2.** Characteristics of FINRISK 2002 participants (N=8726) who took part in the fecal sampling and those who did not.

| Variable | Participated | Did not participate | P value |
| --- | --- | --- | --- |
| Participants, n | 7231 | 1495 |  |
| Men, n (%) | 3248 (44.9) | 703 (51.4) | <0.001 |
| Age, years | 49.5 (13.0) | 40.4 (11.3) | <0.001 |
| BMI, kg/m^2^ | 27.0 (4.7) | 26.5 (4.7) | <0.001 |
| MAP, mmHg | 98.0 (12.7) | 95.6 (12.8) | <0.001 |
| Current smoking, n (%) | 1687 (23.4) | 567 (38.1) | <0.001 |
| Cholesterol, mmol/L | 5.6 (1.1) | 5.4 (1.1) | <0.001 |
| PRS | 0.00 | 0.00 | 0.68 |
| Leisure-time exercise |  |  |  |
| Limited, n (%) | 1510 (21.2) | 456 (30.9) | <0.001 |
| Moderate, n (%) | 4012 (56.3) | 639 (43.3) |  |
| Vigorous, n (%) | 1598 (22.4) | 381 (25.8) |  |

MAP, mean arterial pressure; HDL, high-density lipoprotein; BMI, body mass index; PRS, systolic blood pressure polygenic risk score. Data may be missing for some individuals.

**Supplemental Table S3.** AUROC values of various models for hypertension risk prediction.

| Iteration | C | G | Me | Mi | C+G | C+Me | C+Mi | Mi+G | Mi+Me | Me+G | C+Me+G | C+Mi+Ge | Mi+Me+G | C+Mi+Me | C+Mi+Me+G |
| --- | --- | --- | --- | --- | --- | --- | --- | --- | --- | --- | --- | --- | --- | --- | --- |
| 1 | 0.710 | 0.577 | 0.562 | 0.534 | 0.742 | 0.687 | 0.725 | 0.493 | 0.575 | 0.587 | 0.705 | 0.731 | 0.600 | 0.704 | 0.712 |
| 2 | 0.748 | 0.535 | 0.609 | 0.569 | 0.758 | 0.735 | 0.741 | 0.485 | 0.619 | 0.636 | 0.743 | 0.748 | 0.627 | 0.732 | 0.711 |
| 3 | 0.744 | 0.523 | 0.615 | 0.508 | 0.712 | 0.717 | 0.721 | 0.555 | 0.617 | 0.614 | 0.712 | 0.721 | 0.605 | 0.711 | 0.720 |
| 4 | 0.693 | 0.583 | 0.620 | 0.561 | 0.720 | 0.707 | 0.698 | 0.504 | 0.552 | 0.619 | 0.719 | 0.699 | 0.583 | 0.695 | 0.704 |
| 5 | 0.729 | 0.596 | 0.565 | 0.474 | 0.744 | 0.698 | 0.716 | 0.501 | 0.531 | 0.517 | 0.713 | 0.708 | 0.558 | 0.698 | 0.702 |

Abbreviations: C, Clinical; G, genotype; Me, metabolome; Mi, microbiome.

**Supplemental Table S4.** Association between individual predictors included in the best-performing model (the clinical data with polygenic risk scores) and incident hypertension in men and in women.

|  | Men | | Women | |
| --- | --- | --- | --- | --- |
| Variable | HR (95% CI) | P | HR (95% CI) | P |
| Age, years | 1.53 (1.22—1.91) | <0.001 | 1.66 (1.41—1.96) | <0.001 |
| BMI, kg/m^2^ | 1.46 (1.15—1.86) | 0.002 | 1.49 (1.33—1.66) | <0.001 |
| MAP, mmHg | 1.44 (1.14—1.83) | 0.003 | 1.49 (1.26—1.77) | <0.001 |
| Current smoking, n (%) | 0.88 (0.58—1.34) | 0.55 | 1.58 (1.10—2.25) | 0.012 |
| Healthy food choices score | 0.89 (0.69—1.13) | 0.34 | 0.97 (0.82—1.15) | 0.73 |
| Glucose, mmol/L | 1.11 (1.00—1.24) | 0.06 | 1.19 (0.96—1.47) | 0.12 |
| Polygenic risk score | 1.21 (0.97 —1.50) | 0.10 | 1.29 (1.11 —1.50) | <0.001 |
| Leisure-time exercise, moderate | 0.88 (0.55—1.40) | 0.50 | 0.65 (0.47—0.92) | 0.02 |
| Leisure-time exercise, vigorous | 0.49 (0.26—0.90) | 0.02 | 0.70 (0.43—1.14) | 0.15 |

All variables were simultaneously included in the model. Hazard ratios for continuous variables are reported per 1-SD increase and adjusted for principal components 1-20. Plasma glucose is a 4-hour semi-fasting value. Exercise variables are compared to the lowest class (limited). MAP, mean arterial pressure; BMI, body mass index; PRS, systolic blood pressure polygenic risk score.

**Supplemental Figure S1.** Calibration plots for the agreement between predictions and observations in deciles of the predicted risk obtained from the Cox models with (A) and without (B) the polygenic risk score.


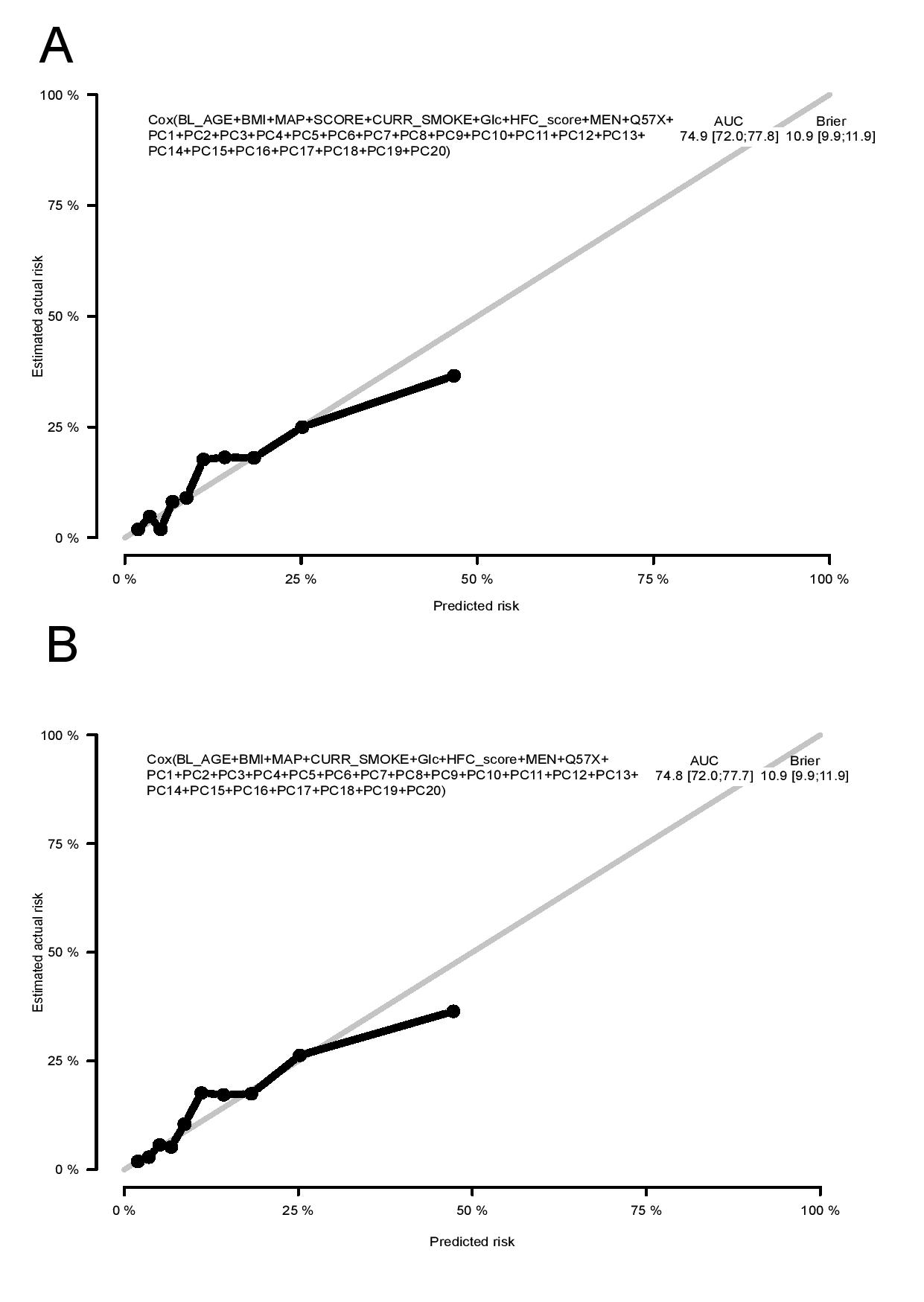

Supplement: Supplementary file 1 [file hyp-83-e25358-s001.docx]
